# Silk Foams with Metallic Nanoparticles as Scaffolds for Soft Tissue Regeneration

**DOI:** 10.3390/ijms252212377

**Published:** 2024-11-18

**Authors:** Claire de Lartigue, Cristina Belda Marín, Vincent Fitzpatrick, Antonella Esposito, Sandra Casale, Jessem Landoulsi, Erwan Guénin, Christophe Egles

**Affiliations:** 1 Univ Rouen Normandie, INSA Rouen Normandie, CNRS, Normandie Univ, Polymères Biopolymères Surfaces (PBS) UMR 6270, 55 Rue Saint-Germain, 27000 Évreux, France; claire.de-lartigue@univ-rouen.fr; 2 Alliance Sorbonne Université, Université de Technologie de Compiègne (UTC), TIMR EA 4297 UTC/ESCOM, CS 60319, 60203 Compiègne, France; cristina.belda-marin@utc.fr (C.B.M.); erwann.guenin@utc.fr (E.G.); 3 Laboratoire de Réactivité de Surface (UMR CNRS 7197), Sorbonne Université, 75252 Paris, France; sandra.casale@sorbonne-universite.fr (S.C.); jessem.landoulsi@sorbonne-universite.fr (J.L.); 4 Department of Biomedical Engineering, Tufts University, Medford, MA 02155, USA; vincent.fitzpatrick@tufts.edu; 5 Alliance Sorbonne Université, Université de Technologie de Compiègne, CNRS, UMR 7338 Biomécanique et Bioingénierie (BMBI), Centre de Recherche Royallieu, CS 60319, 60203 Compiègne, France; 6 Univ Rouen Normandie, INSA Rouen Normandie, CNRS, Groupe de Physique des Matériaux UMR 6634, 76000 Rouen, France; antonella.esposito@univ-rouen.fr

**Keywords:** silk, iron oxide, gold and silver nanoparticles, foam, biomaterial, scaffold, tissue engineering

## Abstract

Tissue regeneration can be achieved by providing endogenous cells with a biomaterial scaffold that supports their adhesion and proliferation, as well as the synthesis and deposition of an extracellular matrix (ECM). In this work, silk fibroin protein foams were formed by lyophilization to generate tissue engineering scaffolds. Three types of medically relevant nanoparticles (NPs) (iron oxide, gold and silver) were added to this biomaterial to assess the ability of silk foams to be functionalized with these NPs. The structural and mechanical properties of the foams with and without the NPs were suitable for tissue support. The in vitro cytocompatibility of the scaffolds was confirmed according to the ISO 10993 guidelines. The biocompatibility of the scaffolds was investigated by assessing inflammation and endogenous cell colonization in a mouse subcutaneous model These in vivo experiments demonstrated a loss of acute inflammation and the absence of chronic inflammation in the grafted animals. The obtained results show that silk foams are good candidates for supporting soft tissue regeneration with the additional possibility of functionalization with NPs.

## 1. Introduction

Diseases, treatments, or injuries can lead to a loss of soft tissues, including skin, nerves, tendons, ligaments and cartilage. Regenerative medicine based on tissue engineering represents a promising strategy to repair such damage, provided that biocompatible scaffolds are available to support cells and tissues. These scaffolds should promote and drive the cells’ regenerative responses towards adhesion, proliferation and extracellular matrix (ECM) deposition, while the material degrades in a predictable, tunable and non-toxic manner.

Numerous biomaterials have been developed with the aim of in vivo utility, but many obstacles to providing optimal biomaterial matrices to support tissue regeneration remain. The physical, chemical, biomechanical and biological properties of scaffolds should match the desired application [1]. Moreover, existing scaffolds can be improved through additional functionalization to address specific requirements [2]. In general, scaffolds should possess mechanical properties that match the surrounding healthy tissue, and have a structure that is compatible with cell infiltration, motility and growth. Functional tissues require specific architectures and mechanical properties for cells to grow and differentiate [3]. Furthermore, it is common to observe an in vivo immune response after the implantation of a biomaterial. However, if this response is persistent (more than two weeks), this can lead to graft rejection [4]. Another common undesired effect observed after implantation is infection, which could result in the need to remove the graft. For these reasons, generating scaffolds that promote proliferation and growth without inducing uncontrolled inflammation is crucial for tissue engineering.

The biocompatibility, mechanical properties and versatility of silk have led to much interest in using it in the medical field, for medical devices (e.g., sutures [5]) but also as a tissue engineering scaffold. FDA approval of certain applications of silk fibroin [6] have encouraged researchers to investigate how this biomaterial could be manufactured into a wide range of constructs, including foams, films, hydrogels, electrospun nanofiber mats, microspheres and additively manufactured objects [7,8].

The current study focuses on foams prepared from silk fibroin, whose porosity can be controlled in terms of its density, size and pore interconnectivity. One of the advantages of foams is that their shape memory features support compression prior to implantation and the subsequent recovery of their initial volume [9]. Foams are mostly used as scaffolds for tissue regeneration [10] or drug delivery [11]. Unlike hydrogels, the high porosity of foams fosters cell colonization of the entire volume [12].

Silk can be functionalized with various types of molecules and nanoparticles (NPs), providing it with a large variety of properties. For instance, antibacterial properties have previously been demonstrated for silver NPs embedded into silk fibroin hydrogels [13]. This property is of special interest for medical devices intended for implantation, to avoid nosocomial infections. This property can also be developed for clothing and textile industry [14]. Silver NPs are also interesting because of their enhanced electrical conductivity due to their surface plasmon resonance effect. This property has been used, for example, for ion sensing [15] and to protect DNA from irradiation damage [16]. The same phenomenon applies to gold NPs; silk fibroin scaffolds containing such NPs were successfully applied for enhancing nerve regeneration when compared to the pristine silk materials [17]. On the other hand, iron oxide and gold NPs are also of special interest for implantable devices as they can be used as contrast agents for medical imaging or as biosensors for an SERS substrate [18]. Gold NPs allow CT or PET scans to be realized, while iron oxide NPs are easily visualized by MRI, thanks to their supermagnetic properties. This specific property has also been previously shown for the iron oxide NPs used within this study [19]. Additionally, these two types of NPs can also be used for hyperthermia treatment [20,21] (stimulating them with a magnetic field and infrared light, respectively) or in phototherapy for iron oxide NPs [22,23].

This work presents a method to functionalize silk fibroin foams with three different metallic NPs, gold (Au NPs), silver (Ag NPs) and iron oxide (Fe NPs). These NPs were chosen as model NPs given their added value for implants (i.e., medical imaging and antibacterial applications). The structural consequences of the addition of NPs to silk fibroin foams were evaluated by microscopic observations and mechanical assessments. The cytocompatibility and the integration of the foams were tested in vitro and in vivo, respectively.

## 2. Results

### 2.1. Structure and Composition of the Foam

#### 2.1.1. Structure

NP synthesis and characterization have been described in previous work [13]. Foam solutions were realized by mixing a silk fibroin solution with either an NP suspension or ultrapure water. The resulting mixtures were then frozen and lyophilized to synthesize the foams.

**Figure 1:**
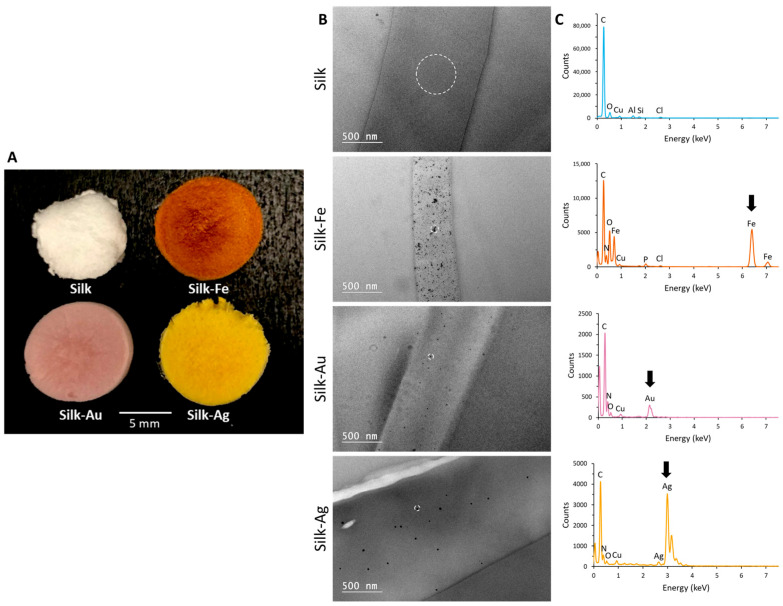
(**A**) Silk foams without and with nanoparticles; (**B**) TEM micrographs of the foams; and (**C**) EDX spectra recorded in the locations indicated by dotted line circles in panel B.

The obtained NP-functionalized foams were first observed at the macroscopic scale, revealing a clear and homogenous coloration that may indicate the presence of well-distributed NPs: brown, pink and yellow for the iron oxide, gold and silver NPs, respectively (Figure 1A). The presence of NPs and their distribution within the silk foam matrix was further examined using Transmission Electron Microscopy (TEM). For this purpose, the NPs-functionalized foams were embedded in a resin and cut into thin slices in a way that allowed the whole thickness (~80 nm) to be probed. The results confirmed the presence of iron oxide, gold and silver NPs in the TEM micrographs (Figure 1B) and EDX spectra (Figure 1C). The NPs were, indeed, shown to be embedded within the silk matrix with an outstanding dispersion, particularly for the gold and silver NPs for which isolated particles are easily discernable (Figure 1B).

**Figure 2:**
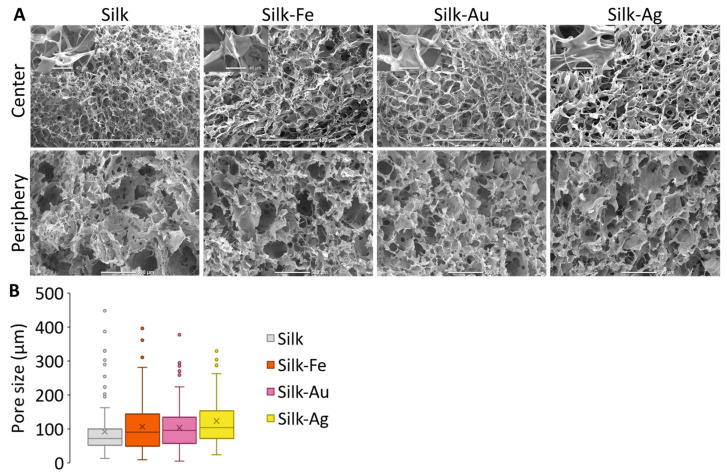
(**A**) SEM images of foams. Scales bars for center: 400 µm, zoom: 40 µm and periphery: 500 µm (**B**) Pore size distribution within foams. No significative statistical difference for *p* < 0.1.

The structures of the foams were observed by using Scanning Electron Microscopy (SEM). Figure 2A shows the morphology of the different biomaterials. All samples presented a highly porous structure with no major modifications to the foam porosity when NPs were added. Furthermore, no NP aggregates were observed, suggesting a homogenous distribution inside the foams. All foams showed a homogeneous porosity with interconnected pores. An analysis of the pore size distribution, shown in Figure 2B, revealed a substantial microporosity, with median pore diameters around 91 µm regardless of foam composition. No statistical difference was measured between the foams.

In order to assess the interconnectivity of the pores, we carried out a dye uptake assay. The blue dye solution was fully absorbed by the foam in a short period of time. The materials were then cut in half to prove the homogeneity of the uptake; indeed, the blue dye was present everywhere inside the foam. The interconnection of pores within the silk foam was demonstrated and the liquid was measured to spread at a speed of 0.77 ± 0.16 mm/s.

#### 2.1.2. Compression Tests

**Figure 3:**
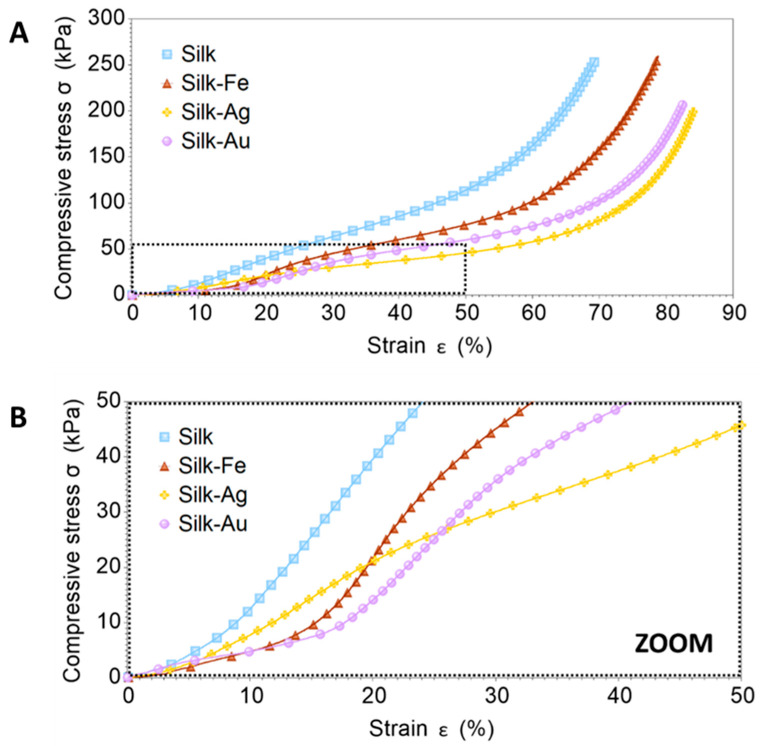
(**A**) Compressive curves recorded for silk, silk-Fe, silk-Ag and silk-Au foams in controlled-force conditions. The force ramp was applied up to 18 N at a rate of 0.5 N/min and 37 °C. (**B**) Zoom in for the initial compression response (up to 50 kPa, 50% compressive strain).

**Figure 4:**
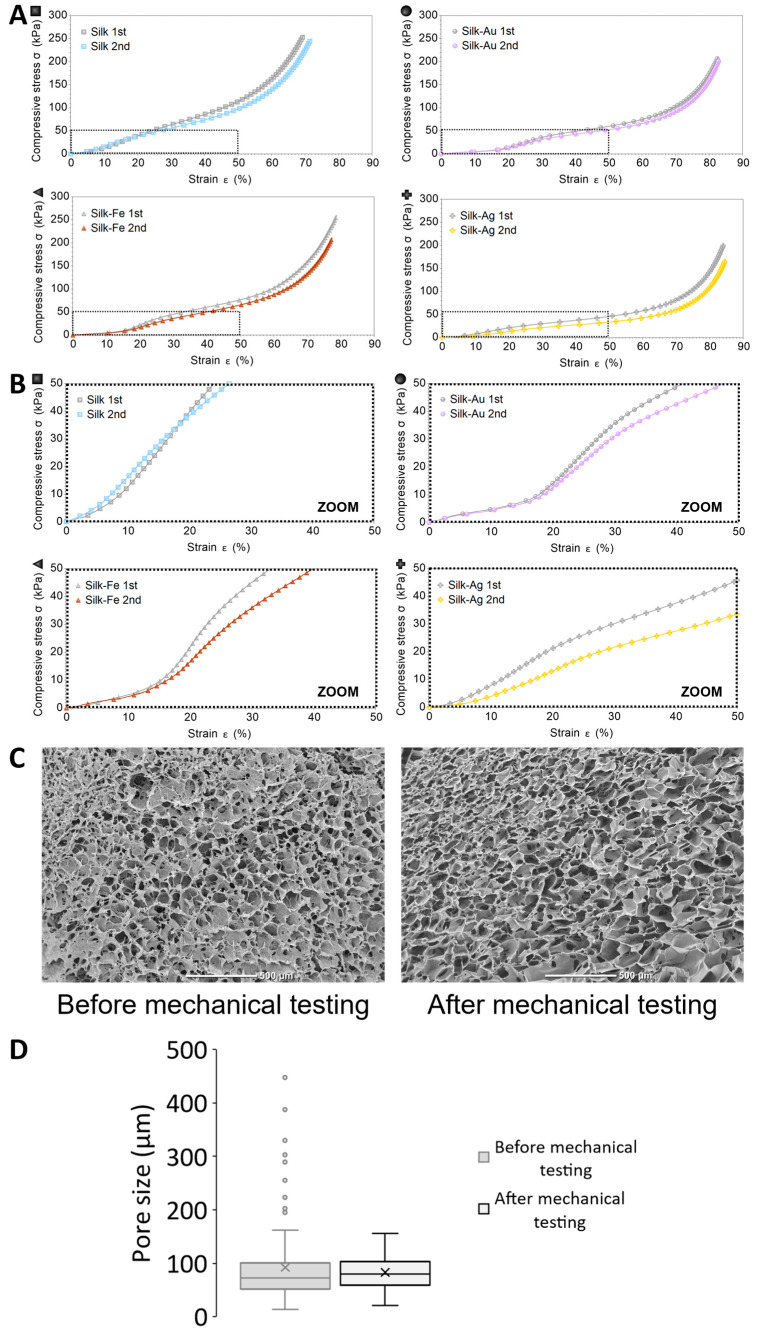
(**A**) Repetition of compression tests as in Figure 3 (same measurement conditions) after a recovery time of about 3 h at room temperature. For each sample, the first compression curve is represented in gray, whereas the second one is represented in colors. (**B**) Zoom in on the initial part of the curves (up to 50 kPa, 50% compressive strain). (**C**) SEM images of silk foams before and after mechanical testing. (**D**) Pore size distribution within silk foam before and after mechanical testing. No significative statistical difference for *p* < 0.1.

The foams were tested under compression (Figure 3) to evaluate dynamic changes in their shape recovery properties. The compression mode was selected because porous biomaterials implanted in living organisms must resist the pressure from surrounding fluids and tissues. The test was repeated after a recovery time of a few hours (Figure 4) as surgeons may need to squeeze the biomaterial for easier implantation, such as to fit into a confined tissue space, but then the biomaterial should recover its shape and mechanical properties to ensure space filling functions, e.g., in the replenishment of cavities and/or support of growing tissues.

Figure 3 shows the compressive mechanical behavior recorded for the silk, silk-Fe, silk-Ag and silk-Au foams. The methods and experimental parameters for testing foams in compression were selected based on a targeted strain rate [24], since the compressive response could be rate-sensitive, especially for closed-cell foams [25]. In this study, the compression tests were intended to reveal consequences of the incorporation of NPs into the matrix of the foamed silk. SEM showed that all the foams had interconnected pores. Thus, the compression tests were performed in arbitrarily selected controlled-force conditions, corresponding to low strain rates (10^−4^–10^−2^ s^−1^).

The compressive deformation of all the foams demonstrated four separate steps, which were particularly visible at high strain rates: the first step was a linear elastic response with strain and deformation with a very small slope; the second step was steeper. These two first steps were fully recoverable due to the elastic bending of cavity edges and the stretching of cavity faces. The third step, also known as the collapse plateau, was flatter where the stress remains constant while deformation increases due to the foam edges undergoing plastic bending or buckling (this phase usually informs the material’s capability to absorb energy). Finally, the fourth step corresponded to densification, where the sample progressively approached the behavior of a compact solid (the cellular structure initially present in the foam is fully collapsed and the cell walls start to interact) [25,26]. All the steps, along with their characteristics (strain range and slope), are summarized in Table 1. In the selected experimental conditions, the incorporation of NPs changed the mechanical response of the foams to compression.

The incorporation of Fe NPs had the most significant effect on decreasing the resistance to compression with small deformations (up to 3%), whereas the Au NPs had no significant effect compared to the pristine (control) silk foams. The presence of both Fe and Au NPs extended the strain range of the first deformation step, but only the Fe NPs had a significant effect on the strain range in the second deformation step (reduced in comparison with the other samples). The strongest resistance to deformation in the second step was from the Fe NPs. All the NPs significantly extended the strain range in the third deformation step but reduced the resistance to deformation (slope). NPs-modified foams support larger deformations at the maximum load (80–85%), whereas pristine (control) foamed silk only deforms up to 70%. The incorporation of NPs improves the compression/recovery of silk foams without degrading the mechanical properties; the slope estimated from the last deformation step (densification) suggests that the NPs effectively reinforced the foamed silks, and that the strongest reinforcement effect was achieved with the Ag NPs. In contrast, the Au and Fe NPs had a more significant effect on changing the mechanisms of compressive deformation, because their incorporation in foamed silk produced a clear change in the shape of the mechanical response, in that the transitions from one step to another were more significant (Figure 3A).

Figure 4 shows that all the foams recovered most of their original compressive behavior a few hours after compression (gray curves represent the mechanical responses to the first force ramp, the color curves represent the mechanical responses to the second force ramp, performed on the same sample in the same experimental conditions). Moreover, the SEM observations did not show any difference before and after mechanical testing (Figure 4C). These results were confirmed with the measurement of the pore size distribution in both foams (Figure 4D), with median pore diameters at 72 µm and 80 µm before and after mechanical testing, respectively. The four-step mechanism of compressive deformation was generally maintained. The preliminary results shown in Figure 4 (full deformation range in panel A, zoom in on the first part of the test in panel B) suggest that the foams containing Fe and Au NPs recovered after compression (at least up to 15–20% strain), whereas the foamed silks containing Ag NPs had the worst recovery. It should be noted that each sample was unloaded between the first and the second test, and that each time new values of diameter and thickness were entered into the software controlling the mechanical tests. Performing quantitative compression tests on foam materials is challenging [27]; therefore, the results reported here should mostly considered be for comparison purposes. However, all the foams (except the pristine ones) recovered their initial shape and dimensions. Thus, the incorporation of NPs slightly reinforced the elastomeric behavior of silk foams, while increasing their densification moduli.

**Figure 5:**
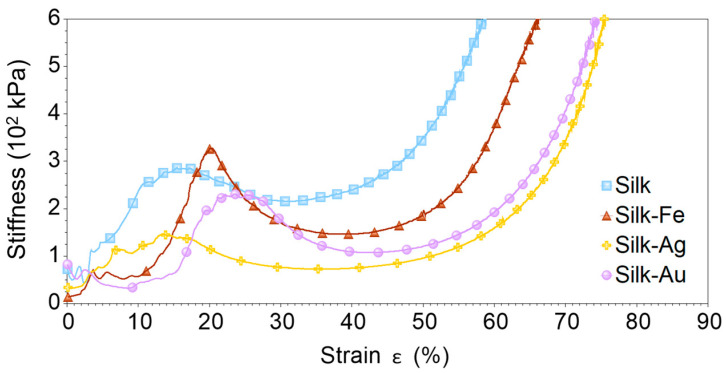
Stiffness (obtained as the derivative of stress vs. strain curves) vs. strain plots obtained for silk, silk-Fe, silk-Ag and silk-Au foams.

The energy absorbed to yield for all the foams was estimated according to the method used by Li and Aspden [28]. The stiffness was calculated as the derivative of the compressive stress vs. strain curves in Figure 3, and then plotted as a function of strain, revealing a stiffness peak (yield) as shown in Figure 5. The energy absorbed to yield was calculated by integrating the compressive strain-stress curves in Figure 3 up to the yield strain; this value can be considered a rough estimation of resilience, which is the energy that a sample can absorb and still return to its original state (elastic deformation). The results summarized in Table 2 confirm that the foams containing Au and Fe NPs had the highest yield strain. Au NPs appeared to be the best choice for energy absorption at low compressive deformations.

#### 2.1.3. Swelling

**Figure 6:**
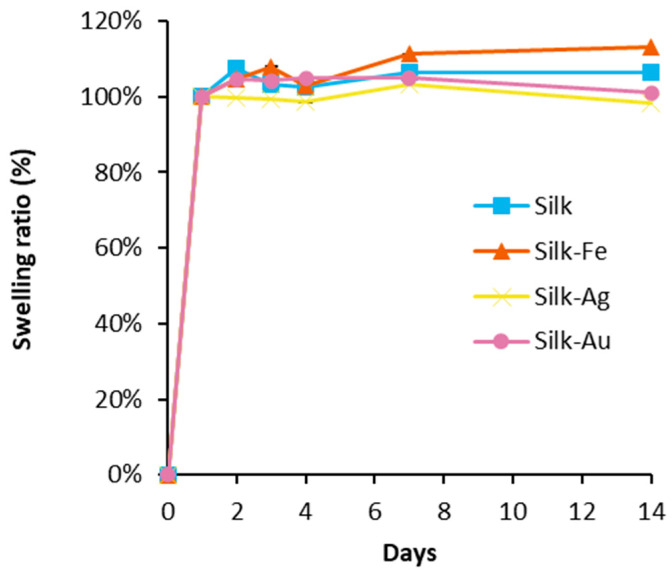
Normalized swelling ratio; standard deviation close to 0.

The water absorption capacity of the foams is predictive of the diffusion of culture medium and nutrients. The swelling behavior is shown in Figure 6. The biomaterials showed rapid swelling, followed by a stable equilibrium which was reached in one day and then stabilized. No significative differences were observed between the four systems. A high-swelling foam is a consequence of its porous structure, allowing liquid retention to assist as a nutrient reserve for the cells.

### 2.2. Biological Behavior

#### 2.2.1. Viability

**Figure 7:**
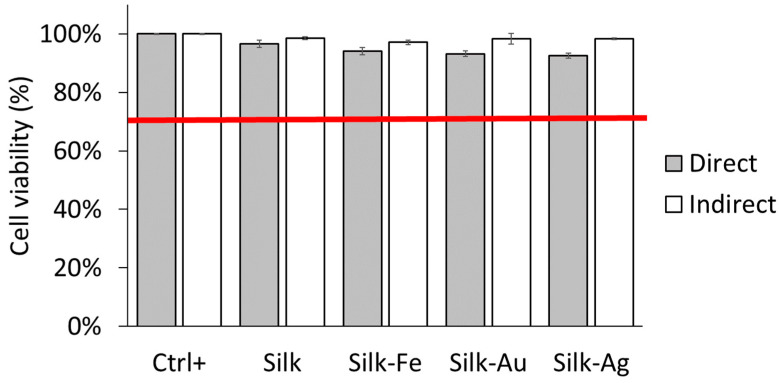
Viability measured for silk and silk-NPs foams. Cells in culture medium were used as a positive control. Red line is the threshold (70%) given by the ISO 10993-5:2009 standard No statistical difference was observed [29].

Biocompatibility and the absence of cytotoxicity are important to check for a biomaterial intended for implantation. Murine fibroblasts (L929 cell line) were cultured for 24 h either directly on disinfected foams or indirectly in conditioned medium that had been in contact with the foams overnight. Both tests were carried out following the ISO 10993-5 recommendations [29]. Cell metabolism was used as an indicator of viability, and was assessed using an AlamarBlue^®^ test. Figure 7 shows that in either direct or indirect contact with the foams, the cells maintained a high viability (>80%) which was higher than the threshold (70%) requested by the ISO 10993-5:2009 standard [29]. No statistical difference was found. Therefore, no cytotoxic effects were observed for any of the four biomaterials.

#### 2.2.2. Cell Adhesion and Spreading

**Figure 8:**
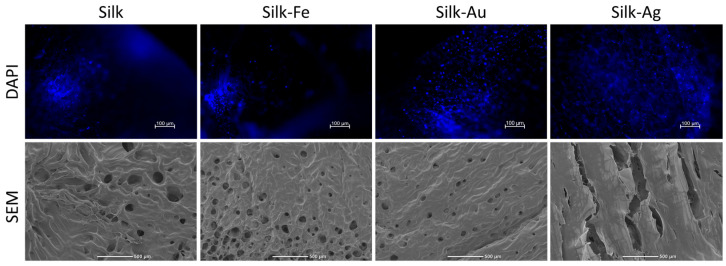
Foams cultured with cells observed under a florescent microscope with DAPI staining and with SEM.

A cell culture of L929 murine fibroblasts on the foams was carried out for 96 h, and after a DAPI staining were observed under a fluorescence microscope and SEM. Figure 8 shows that cells adhered to the foams. No difference was observed in the presence of NPs. Cells were also observed inside the foams demonstrating that they were able to colonize the bulk of the biomaterial.

#### 2.2.3. Irritation and Inflammatory Response

**Figure 9:**
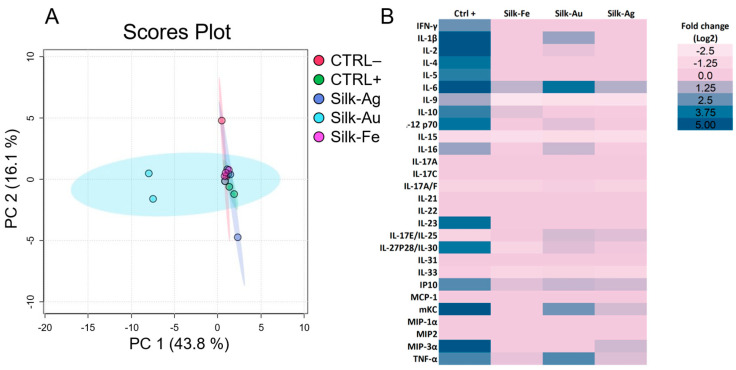
(**A**) PCA analysis of the secretome of macrophages cultured on the silk and silk-NP foams for both untreated (CTRL−) and treated (CTRL+) controls. (**B**) J774.2 murine macrophage inflammatory secretome upon exposure to silk or silk-NP foams.

Irritation is the first step in an inflammatory response, characterized by the secretion of IL-1, IL-6 and TNF-α. Here, a murine J774.2 macrophage cytokine secretome heatmap (Figure 9B) was generated after a 24 h exposure to the different foams in comparison to untreated (CTRL−) and pro-inflammatory LPS-treated (CTRL+) controls to assess cytocompatibility. This heatmap highlights an expected acute inflammation induced by the addition of 2 µg/mL LPS with a significant secretion of numerous pro-inflammatory cytokines. The pro-inflammatory response was also evidenced via principal component analysis (PCA, Figure 9A), as the LPS-treated condition represents a cluster isolated from the untreated condition. Foam samples did not induce the secretion of pro-inflammatory cytokines and chemokines to the same extent as the positive control (LPS), as shown by the heatmap profiles. Only foams containing gold NPs induced IL-6 and TNF-α secretion.

#### 2.2.4. In Vivo Implantation and Histology

**Figure 10:**
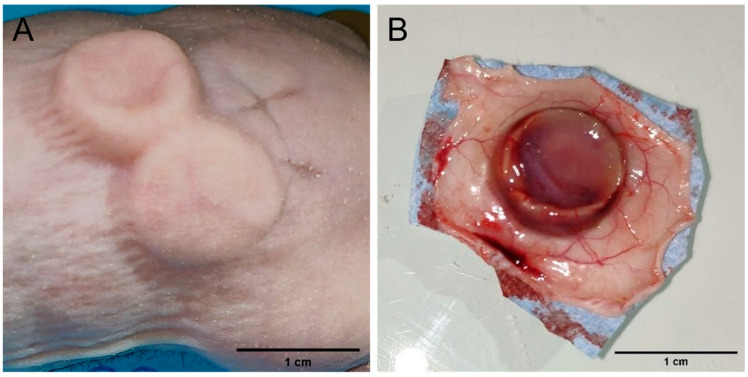
(**A**) Mouse implanted with 2 foams. (**B**) Silk implant 1 month after implantation.

**Figure 11:**
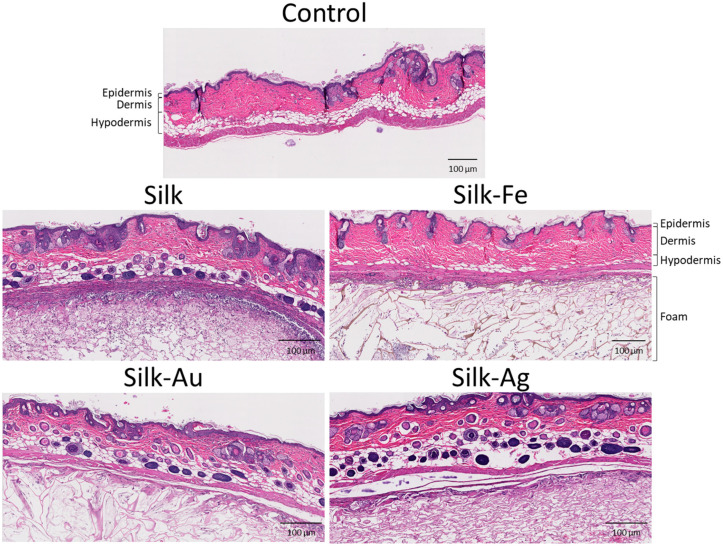
Histological section of implants (20×) in nude mice after 1 month of implantation. Hematoxylin and Eosin coloration.

The biocompatibility of the biomaterials was tested in vivo in nude mice. Foams were implanted subcutaneously (Figure 10A) and observed 1 month later. All the animals survived the implantation without showing signs of discomfort or pain. Necropsy and histological analyses of the biopsy specimens were performed and the results are displayed in Figure 10 and Figure 11, respectively. During the in vivo experiments, the foam structure was maintained for one month after implantation with the material still visible under the skin of the animal (Figure 10A) and with the size of the foam being preserved since the implantation date. Integration of the foams was observed as the implants exhibited a tissue-like structure penetrated by surrounding blood vessels. The vascularization was obtained by sprouting of the surrounding blood vessels as shown on Figure 10B.

Histological analyses of the implanted foam material specimens (Figure 11) showed the accumulation of thick newly formed tissue surrounding the implant. The tissues showed inflammation one month after implantation limited to the surface of the material. The inside of the foam was clear, with extracellular matrix, infiltrated cells, and the absence of eosinophils, neutrophils and macrophages or other remaining inflammatory cells (Figure 11). Tissue synthesis and vascularization at 1-month post-implantation suggests the biocompatibility of the material and its capacity for soft tissue augmentation.

**Figure 12:**
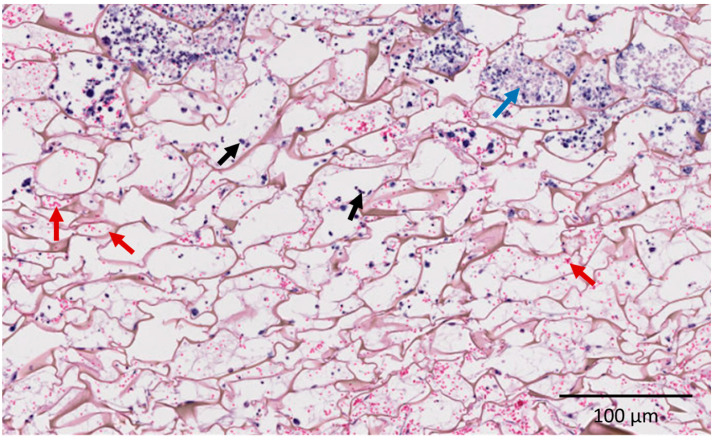
Histological section of implants in nude mice after 1 month implantation. Hematoxylin and eosin coloration. Red arrows: blood vessels; black arrows: cells; blue arrow: limit of inflammatory area.

The homogeneously cellularized tissues showed hematoxylin and eosin staining, with vascularization within an intricate network of blood vessels (Figure 12). The mesenchymal cells were surrounded by extracellular matrix. The remaining area of inflammation was only evident in the outer regions of the implants.

## 3. Discussion

Currently, most research into soft tissue augmentation is focused on developing scaffolds to support cell migration and proliferation. These scaffolds should be biocompatible but also easy to handle during implantation. The development of such a scaffold requires the optimization of composition, morphology, mechanics and chemistry. These scaffolds can be used in various applications ranging from soft to hard tissues. The intended application is often chosen based on the foam’s biomechanical properties. Based on the values reported by Li and Aspden [28], we observed that the foams had an elastic and plastic deformation threshold 10 times higher than that of bone, and a threshold of stress and resilience 100 times lower. According to this work, our silk foams offer potential utility in soft tissue regeneration.

Our results demonstrated the possibility of synthetizing silk foams containing three different types of NPs. These NPs were chosen as model NPs; however, the integration of other NPs may also be interesting depending on the targeted application of the scaffold. Observations of the foams demonstrated a homogeneous distribution of iron oxide, gold and silver NPs within the foams, as depicted by the coloration seen in Figure 1A. The absence of aggregates under SEM reinforces this point. Therefore, we can hypothesize that, if NPs were to be functionalized with molecules such as growth factors, the presence of these molecules will be homogeneous throughout the foam.

The inclusion of NPs did not affect the foam formation procedure, and the foam structures had a controlled and uniform porosity in all conditions. An analysis of the porosity demonstrated that the pore size of the foams was around 150 µm, allowing enough space for cells to colonize the entire biomaterial.

Moreover, the release of these nanoparticles was previously evaluated on similar fibroin scaffolds, and it was found that even after 6 weeks of immersion in water no nanoparticle release was evident and only traces (62.5 ppb) of metal could be detected in water [13].

In previous work, Akturk et al. [30] demonstrated the absence of modifications of the Young Modulus following the adding of gold NPs in silk electrospun dry scaffold. However, in our experiments, the incorporation of NPs in the foams improved the compression capacity of the foams and reinforced elastomeric behavior. This demonstrates that by changing the structure of the material, the incorporation of gold, but also iron and silver NPs in our experiment, can help to tune the biomechanical properties of the material to better fit the final application.

The ability to recover their original compressive behavior in a few hours was also demonstrated for our biomaterials. This shape recovery was due to residual stress, as described by Tcharkhtchi et al. [31]. Due to this shape memory, the biomaterial offers a useful implantability as it can be introduced to the body compressed and can then adapt to the defect geometry. The development of shape memory scaffolds offers many new applications in the biomedical field including reconstructive medicine [32] or the control of cell fate in tissue engineering [33]. It can also be incorporated into a minimally invasive approach to soft tissue reconstruction surgeries.

To assess the cytocompatibility of the foams, and in accordance with relevant ISO standards (ISO 10993-5:2009—Biological Evaluation of Medical Devices [29]), L929 murine fibroblasts were used for in vitro cytotoxicity studies. Our silk biomaterials displayed a good support of cell viability (over 70%), with or without NPs, and were a good scaffold for the growth and proliferation of L929 cells as they adhered to the surface of the scaffolds.

A controlled inflammatory response to scaffold implantation is a critical criterion for success in tissue engineering applications. Therefore, the in vitro quantification of pro-inflammatory cytokines and chemokine secretions was performed by exposing the foams to murine macrophages, which play a key role in the immune response [4]. The results showed that only the foams containing Au NPs induced a limited secretion in IL-6 and TNF-α. This limited inflammation after implantation should help the regeneration of tissues if its controlled and not persistent [34]. The in vitro results therefore suggest that all the foams are suitable for in vivo implantation.

In vivo biocompatibility was further evaluated by the subcutaneous implantation of foams in mice. The presence of a neo-synthesized extracellular matrix and vascularization inside the implants was observed, suggesting integration of the biomaterial into the tissue to facilitate cell colonization, providing enough nutrients and oxygen through the entire foam. As expected, a limited presence of inflammatory cells was observed [35], and combined with the neo-synthetized ECM and vascularization, we can conclude that the biocompatibility of the foams was optimal.

No visual degradation of the foams was observed 1 month after implantation. Nevertheless, silk is known to be biodegradable [36]. A slow degradation of the biomaterial allows the cells that repopulated the silk foam to modify their microenvironment. Biomaterials aimed towards tissue regeneration or reconstruction should degrade at a rate that either matches or is slower than that of tissue regeneration. This is crucial to maintain the overall function, structure and volume of the tissue throughout the healing process, ensuring a complete recovery of the defect. Further studies should be conducted to determine the degradation kinetics of these scaffolds in vivo.

The potential of combining NPs and silk has been shown in the literature [19]. The large versatility of the combinations comes from the structure of the polymer (as a gel, an electrospun fiber or a film) as well as the type of NP. In the literature, most research focuses on silk fibers with added gold nanoparticles [37]. For example, gold nano-composites have been used for nerve regeneration [17] together with electrospun silk fibers, creating a mat to form conduits for nerve regeneration. The authors were able to use the electrical conductivity of the gold NPs to enhance nerve regeneration. In our results, the good distribution of the NPs we observed under Transmission Electron Microscopy (TEM) could also allow electrical-based stimulation for soft tissue augmentation. Other studies describe the same types of NP in electrospun fibers for the culture of cardiomyocytes and mesenchymal stem cells for myocardial regeneration and repair [38]. The authors use the NPs to improve biomaterial imaging. In our results, we use other types of cells but the good biocompatibility towards L929 and later fibroblasts and endothelial cells of the grafted host suggest broader applications, including for cardiac tissues. Previous studies [39] have shown the potential of using electrospun silk fibers as a dressing for wound healing. The addition of gold as well as other metallic nanoparticles could therefore add new potential for wound healing, as described in the literature [13,40].

Moreover, Gold NPs are already used in the medical field as a contrast agent for computed tomography (CT) and photothermal therapy due to the local increase in temperature through laser irradiation [41]. Iron oxide NPs are also used in hyperthermia therapies for cancer, using the application of an external magnetic field [42]. They are also employed as contrast agents in magnetic resonance imaging (MRI) [43]. In addition, the magnetic properties of iron oxide NPs can be used to mechanically stimulate cells [44].

Finally, another range of functionality can be brought to the scaffolds presented in this study by functionalizing the NPs. This can be easily carried out through click chemistry thanks to the presence of HMBP-C≡CH molecules in the NPs surface. Such functionalization can be applied, for example, to the coupling of fluorophores, catalysts, drugs and peptides [45,46]. The covalent bonding of such molecules to the structure of the scaffold proposed herein could avoid the burst effect that is frequently observed for drug delivery systems. The release of the desired molecule could be fine-tuned by tuning the degradation rate of the scaffold, instead of relying solely on the diffusion of the molecule out of the scaffold.

## 4. Materials and Methods

### 4.1. Foam Production

#### 4.1.1. Silk Extraction

Five grams of Bombyx mori cocoons were cut into small pieces and plunged into 2 L of a 0.02 M sodium carbonate boiling solution for 10 min to remove the sericin and obtain fibroin fibers, that were then rinsed in distilled water and dried overnight at room temperature. The dried fibers were placed in a 9.3 M lithium bromide (Sigma-Aldrich, Saint Quentin Fallavier, France, 213225) solution to obtain a 20% (*w*/*v*) silk fibroin solution and the fibroin had a molecular weight between 171 and 460 kDa [40]. The fibroin solution was left to dissolve in an oven at 60 °C for 4 h. The resulting solution was inserted into pre-hydrated 12 mL dialysis cassettes (Thermo scientific, Courtaboeuf, France 66110, MWCO at 3500), and then dialysis was performed against 1 L of ultrapure water with a total of 6 water changes over a period of 3 days. The dialyzed solution was centrifuged twice at 12,700× *g* at 4 °C for 20 min to remove impurities.

#### 4.1.2. Nanoparticle Solutions

##### Gold Nanoparticles (Au NPs)

Au NPs solutions were synthesized following the protocol established in a previous study [19]. Here, 250 µL of hydrogen tetrachloroaurate (III) (HAuCl_4_, 20 mM) and 500 µL of 1-hydroxy-1-phosphonohept-6-ynyl) phosphonic acid (HMBP-C≡CH) solution (40 mM, pH = 10) were added to 19 mL of milliQ water. Then, 55 μL of sodium ascorbate (17.6 mg/L) was added while the solution was vigorously stirred for 30 min. The obtained solution was then dialyzed (molecular weight cut off at 100 kDa) to remove unreacted materials, and stored at 4 °C.

##### Silver Nanoparticles (Ag NPs)

The synthesis of Ag NPs was very similar to the procedure used for the Au NPs. Here, 11.76 μL of silver nitrate (Alfa Aesar, AgNO_3_, 850 mM) and 1 mL of HMBP-C≡CH solution (40 mM, pH = 10) was added to 19 mL of milliQ water. Then, 110 μL of sodium ascorbate (17.6 mg/L) was added before heating using a microwave reactor (Monowave 300, Anton Paar GmbH, Graz, Austria). The reactor was programmed to reach 100 °C, hold this temperature for 15 min and then cool to 55 °C. The mixture was continuously stirred (1200 RPM) during the reaction. The obtained solution was then dialyzed (molecular weight cut off at 100 kDa) to remove unreacted materials, and then stored at 4 °C.

##### Iron Oxide Nanoparticles (Fe NPs)

An alkaline co-precipitation method was used to produce Fe NPs. FeCl_2_·4H_2_O, 0.01 mol (Sigma-Aldrich, Saint Quentin Fallavier, France) was dissolved in 7.5 mL of hydrochloric acid (HCl, 1 M). At the same time, FeCl_3_·6H_2_O (0.02 mol) was dissolved in 160 mL of water. An Fe^2+^/Fe^3+^ solution was prepared by mixing the previous solutions in an ultrasonic bath. A peristaltic pump set at 400 mL/min was used to pour the ferrous solution onto a stirred (2000 RPM) reactor at 30 °C filled with 84 mL of sodium hydroxide solution (NaOH, 2 M). After 2 h, the remaining NaOH was neutralized with hydrochloric acid (HCl, 2.5 M). The pH was then set at 7. Fe NPs precipitation was performed with neodymium magnets and rinsed with water. This process was repeated three times. HCl (1 M) was added to decrease the pH to 2 to stabilize the Fe NPs, which were then stored at 4 °C. An HMBP-C≡CH coating was applied onto the Fe NPs (~0.2 M) by mixing them with a HMBP-C≡CH solution (0.34 × 10^−6^ M, pH = 2) at a 1:1 ratio *v*/*v*. After 2 h of stirring, the suspension was sonicated for 30 min. The NPs were precipitated using a magnetic field and then placed in HCl (10^−2^ M). This process was repeated three times. The Fe NPs were suspended in water at pH = 7 and stored at 4 °C.

As described by Belda-Marin et al. [13], the spherical NPs showed mean diameters of 4.7 ± 1.2 nm, 23.3 ± 1.2 nm and 7.0 ± 1.3 nm for gold, silver and iron, respectively.

#### 4.1.3. Foam Formation

The foams were produced starting from a solution prepared with slow stirring by mixing ultrapure water with 70% glycerol (0.01 g/mL) and a silk fibroin solution (0.03 g of silk fibroin/mL). A multiwell plate was filled with the solutions and then placed at −20 °C overnight. The foams were then produced by lyophilization. The protocol used to produce foams with NPs was similar to prior processes, with the difference that the water was replaced with the NPs solutions (0.5 mM for Fe NPs, 0.25 mM for Au NPs or 0.5 mM for Ag NPs), as previously described [19].

### 4.2. Characterization

#### 4.2.1. Mechanical Properties

Unconfined uniaxial compression tests were performed by a Dynamic Mechanical Analysis (DMA) Q850 (TA Instruments, Guyancourt, France) using a 15 mm-diameter compression clamp with cylinder-shaped specimens (9.2 ± 0.7 mm in diameter, 4.8 ± 0.5 mm in thickness). Two batches of a few samples for each condition were tested to ensure repeatability. Prior to testing, the equipment was fully calibrated (force, clamp mass and compliance), the average diameter of each sample was estimated by repeated measurements with a caliper (measurement error 0.4%), and the thickness of each sample was measured using the built-in function of TRIOS Software (TA Instruments, version 5.1.1.46572). The tests were performed in controlled-force conditions. The temperature was equilibrated at 37 °C for 5 min, then a force ramp was applied up to 18 N at a rate of 0.5 N/min. The same protocol was repeated on the same sample after a recovery time of about 3 h at room temperature.

#### 4.2.2. Swelling Ratio

The water absorption capacity of the scaffold was assessed. The initial dry weight of biomaterials was noted ($m_{dry}$). After hydration by immersion in water at room temperature, each foam was weighed at days 1, 2, 3, 4, 7 and 14 after the removal of excess water, and the swelled weight ($m_{wet}$) was recorded. The water was changed every two days. Triplicates were performed and the swelling ratio was determined using the following equation:

$$Swelling\;ratio=\frac{m_{wet}-m_{dry}}{m_{dry}}\times 100$$

#### 4.2.3. Pores Interconnectivity Measurement

We mixed 3 mL of water with one drop of a blue dye. Then, 250 µL of this solution was placed on a Petri dish. Each foam size was first measured with a digital caliper and was then carefully put on the dye. The time needed for the dye to fully penetrate the foam was measured and the speed was calculated.

#### 4.2.4. Transmission Electron Microscopy (TEM)

TEM experiments were performed using a JEOL-JEM 2100Plus electron microscope (JEOL, Croissy-sur-Seine, France) operating at 200 keV (LaB6 gun) with an Orius camera 4K (Gatan, Pleasanton, CA, USA). EDX spectra were recorded with an SDD 80 mm^2^ detector (Oxford Aztec software, https://nano.oxinst.com/products/aztec/, accessed on 1 August 2024). Silk foam samples were cut into small pieces and embedded in agar resin which polymerized at 70° for 48 h. The obtained samples were then cut into slices with a thickness of approximately 80 nm using an ultramicrotome. The slices were recovered from the surface of the water, by capillary, on copper-mesh TEM grids coated with an amorphous carbon film.

#### 4.2.5. Scanning Electron Microscopy (SEM)

The morphologies of the silk foams, including the pore sizes and pore distributions, were determined using SEM (TESCAN Vega 3 LMU, Brno, Czech Republic). Prior to SEM observations, some foams were cut in half with a scalpel and dried under vacuum and sputter-coated with gold (Q150RS, Quorum technologies, Puslinch, ON, Canada). SEM observations were performed using an Everhart-Thornley detector under a high vacuum with an accelerating voltage of 5 kV and working distances ranging from 6 to 8 mm. The pore size distribution and overall porosity were analyzed with the ImageJ software (version 1.54f) on different SEM images (*n* = 3) from a vertical cross-section of the foams.

### 4.3. In Vitro Test

#### 4.3.1. Viability Test

Foams were disinfected in a bath of absolute ethanol (Fisher Scientific, Illkirch, France E/0600DF/17). Once dried, the foams were placed in 12-well plates and covered with 2 mL of culture medium overnight. Both direct and indirect cell contact techniques were performed. For direct contact, Murine fibroblast, L929 cells (American Type Culture Collection ATCC, Molsheim, France, CCL-1™) were seeded on the foams at 20,000 cells/cm^2^. For the indirect contact method, the foams were put in Alpha MEM Eagle medium (PAN Biotech, Aidenbach, Germany, P04-21050) overnight. Then, 500 µL of supernatant for each sample was put in a new plate and the cells were seeded at the same concentration. A culture medium was added, and the plates were placed in the incubator (37 °C, 5% CO_2_) for 24 h.

A reaction medium, composed of AlamarBlue^®^ (Bio-Rad, Hercules, CA, USA, BUF012B) diluted at 1/10 in culture medium composed of Alpha MEM (Corning™, Samois-Sur-Seine, France 15-012-CV) supplemented with fetal bovine serum (PAN Biotech, Aidenbach, Germany, T30-3306W1), was prepared and heated at 37 °C. The culture medium was removed from the wells with the cells and replaced by the reaction media. The cells were incubated (37 °C, 5% CO_2_) for 3 h, the supernatants were collected for each sample and analyzed in a microplate reader (BioTek^®^, Santa Clara, CA, USA, SynergyTM 2) at 570 and 600 nm.

#### 4.3.2. DAPI Staining

Foams used for the viability test were rinsed twice with Dulbecco’s Phosphate-Buffered Saline (DPBS; Sigma-Aldrich, Saint Quentin Fallavier, France, D8537) and then stained with a DAPI solution (Sigma, D8417) at a final concentration of 7.1 µM for 10 min. Foams were observed with a fluorescent microscope (Axio Scope A1, Zeiss with camera Axiocam 202 mono, Rueil Malmaison, France).

#### 4.3.3. Scanning Electron Microscopy (SEM)

L929 cells were seeded at 50,000 cells/cm^2^ on the disinfected and dried foams. Alpha MEM Eagle medium (PAN Biotech, P04-21050) was added, and the plate was placed in the incubator (37 °C, 5% CO_2_) for 24 h. After 24 h of cell culture, the foams were rinsed with Dulbecco′s Phosphate-Buffered Saline (DPBS; Sigma, D8537), then fixed with a 2.5% glutaraldehyde solution and then successive ethanol baths (50%; 70%; 80%; 90% and 100%). The foams were allowed to dry again under a laminar flow hood for 24 h and sputter-coated with a gold layer using a sample metallizer (Cressington, 108 auto Sputter Coater, Watford, UK). Foams with cells were observed with a high vacuum scanning electron microscope (Jeol, JCM-6000).

#### 4.3.4. Metabolic Activity

The inflammatory pattern of the J774 murine macrophage lineage (ECACC-85011428) was determined after 24 h of indirect exposure to foam extracts. J744 macrophages (American Type Culture Collection ATCC) were seeded at a density of 200,000 cells per well in 12-well plates and incubated overnight. Negative and positive control conditions were generated through exposure to a culture medium alone or with a concentration of 20 µg/mL of lipopolysaccharide (LPS). Supernatants were recovered the following day and stored at −20 °C. The supernatant cytokine and chemokine concentrations (pg/mL) were quantified using the V-plex Proinflammatory Panel 1, Cytokine Panel 1 and Th17 Panel 1 kits from Meso Scale Diagnostics (MSD, Rockville, MD, USA) according to the manufacturer’s instructions. These values were replaced by the threshold values for concentrations below or above the detection limits. The concentrations were expressed as a log2-fold change from the untreated control. To identify the main axes of variance and different patterns within this multidimensional data set, the data were processed under principal component analysis following an autoscaling normalization using Metaboanalyst software (version 6.0) [47].

### 4.4. In Vivo Tests

All animal experiments were performed in compliance with European Directive 2010-EU63 [48] and the ARRIVE guidelines [49]. The study design, the sample size, the outcomes and the experimental procedures were approved by the “Comité Régional d’Ethique en Matière d’Expérimentation Animale de Picardie” (CREMEAP; C2EA-96). The chosen experimental animals were pathogen-free 5-week-old male athymic mice (Rj:NMRI-Foxn1nu/nu, 30 g, JANVIER LABS, Le Genest-Saint-Isle, France). Animals were housed in polycarbonate cages in a temperature- and humidity-controlled room and had food and water ad libitum. The 70%-ethanol-disinfected foams were implanted subcutaneously on the backs of athymic mice. The animals were euthanized after one month, and their back skin was harvested, observed under a microscope, and processed using classical histology procedures (Althisia, Troyes, France) for an anatomopathological readout by a certified professional. Non-operated mice were considered negative controls.

#### Histology

Histology techniques (trimming, embedding and H&E tissue sections) and slide scanning were performed at Althisia laboratories (Troyes, France). Histopathological analysis was performed at Sciempath Bio (Lillois, Belgium). The histopathological evaluation of the local effects at the implantation site was based on ISO 10993-6 [50]. In addition to the evaluation of the inflammatory reaction, the amount of foreign material debris and of its integration within the dermal tissue was evaluated.

## 5. Conclusions

Tissue engineering strategies to promote the growth of soft tissue are widely studied to produce scaffolds. Foams have been developed for this purpose. Silk fibroin, a natural polymer with biodegradable and biocompatible properties, was used to create foams with metallic NPs embedded into them. Our study demonstrated that the structure and biocompatibility of these foams were not impacted by the addition of gold, silver or iron oxide NPs. Moreover, the vascularization and the integration of foams in vivo proved that this approach enables the fabrication of biomaterials able to maintain cells alive and to promote soft tissue regeneration.

The successful addition of three types of NPs into the silk foams, without aggregation of the NPs or silk structure changes, opens up a new world of applications derived from the NPs’ intrinsic properties. To go further, it would now be interesting to functionalize NPs with molecules that would improve foam integration or promote the development of soft tissues, such as growth factors. For this purpose, further studies need to be conducted to assess if an interaction between silk and NPs occurs and evaluate the fate of NPs after scaffold degradation.

By exploiting its properties and the possibility of functionalizing biomaterials, it is possible to develop scaffolds that serve the needs of the biomedical field for implantation purposes.

## Figures and Tables

**Table 1:** Steps of compressive deformation observed in silk, silk-Fe, silk-Ag and silk-Au foams in controlled-force conditions (force ramp up to 18 N at a rate of 0.5 N/min and 37 °C). For each step, the strain range and the slope (calculated as the engineering stress on the strain in the corresponding strain range) are provided. The limit between steps was evaluated as the intersection of the corresponding slopes (onset for slope change).

		Silk	Silk-Fe	Silk-Ag	Silk-Au
Step #1	Strain range (%)	0–5	0–15	0–5	0–17
	Slope (kPa) first 3%	60	20	36	60
Step #2	Strain range (%)	5–20	15–25	5–20	17–30
	Slope (kPa)	260	320	140	225
Step #3	Strain range (%)	20–60	25–70	20–75	30–75
	Slope (kPa)	230	150	76	110
Step #4	Strain range (%)	60–70	70–80	75–85	75–85
	Slope (MPa) last 5%	1.28	1.43	1.56	1.40

**Table 2:** Yield strain ε_y_, yield strength σ_y_ and energy absorbed to yield U_r_ for silk, silk-Fe, silk-Ag and silk-Au foams.

	Silk	Silk-Fe	Silk-Ag	Silk-Au
$\varepsilon_{y}$ (%)	15 ± 1	20 ± 1	15 ± 2	25 ± 4
$\sigma_{y}$ (kPa)	28 ± 3	22 ± 10	15 ± 1	23 ± 1
$U_{r}$ (J m^−3^)	272 ± 50	194 ± 50	110 ± 20	324 ± 60

## Data Availability

The original contributions presented in the study are included in the manuscript and figures; further inquiries can be directed to the corresponding authors.

## References

[1] Chen D.X.B., Chen D.X.B. (2019). Scaffold Design. Extrusion Bioprinting of Scaffolds for Tissue Engineering Applications.

[2] Makvandi P., Iftekhar S., Pizzetti F., Zarepour A., Zare E.N., Ashrafizadeh M., Agarwal T., Padil V.V.T., Mohammadinejad R., Sillanpaa M. (2021). Functionalization of polymers and nanomaterials for water treatment, food packaging, textile and biomedical applications: A review. Environ. Chem. Lett..

[3] Eltom A., Zhong G., Muhammad A. (2019). Scaffold Techniques and Designs in Tissue Engineering Functions and Purposes: A Review. Adv. Mater. Sci. Eng..

[4] Boehler R.M., Graham J.G., Shea L.D. (2011). Tissue engineering tools for modulation of the immune response. BioTechniques.

[5] Thilagavathi G., Viju S., Basu A. (2015). 11—Silk as a suture material. Advances in Silk Science and Technology.

[6] Zhang W., Chen L., Chen J., Wang L., Gui X., Ran J., Xu G., Zhao H., Zeng M., Ji J. (2017). Silk Fibroin Biomaterial Shows Safe and Effective Wound Healing in Animal Models and a Randomized Controlled Clinical Trial. Adv. Healthc. Mater..

[7] Rockwood D.N., Preda R.C., Yücel T., Wang X., Lovett M.L., Kaplan D.L. (2011). Materials fabrication from Bombyx mori silk fibroin. Nat. Protoc..

[8] Sang Y., Li M., Liu J., Yao Y., Ding Z., Wang L., Xiao L., Lu Q., Fu X., Kaplan D.L. (2018). Biomimetic Silk Scaffolds with an Amorphous Structure for Soft Tissue Engineering. ACS Appl. Mater. Interfaces.

[9] Brown J.E., Moreau J.E., Berman A.M., McSherry H.J., Coburn J.M., Schmidt D.F., Kaplan D.L. (2017). Shape Memory Silk Protein Sponges for Minimally Invasive Tissue Regeneration. Adv. Healthc. Mater..

[10] Ornell K.J., Taylor J.S., Zeki J., Ikegaki N., Shimada H., Coburn J.M., Chiu B. (2020). Local delivery of dinutuximab from lyophilized silk fibroin foams for treatment of an orthotopic neuroblastoma model. Cancer Med..

[11] Chambre L., Parker R.N., Allardyce B.J., Valente F., Rajkhowa R., Dilley R.J., Wang X., Kaplan D.L. (2020). Tunable Biodegradable Silk-Based Memory Foams with Controlled Release of Antibiotics. ACS Appl. Bio Mater..

[12] Bellas E., Lo T.J., Fournier E.P., Brown J.E., Abbott R.D., Gil E.S., Marra K.G., Rubin J.P., Leisk G.G., Kaplan D.L. (2015). Injectable Silk Foams for Soft Tissue Regeneration. Adv. Healthc. Mater..

[13] Belda Marín C., Egles C., Humblot V., Lalatonne Y., Motte L., Landoulsi J., Guénin E. (2021). Gold, Silver, and Iron Oxide Nanoparticle Incorporation into Silk Hydrogels for Biomedical Applications: Elaboration, Structure, and Properties. ACS Biomater. Sci. Eng..

[14] Lu Z., Xiao J., Wang Y., Meng M. (2015). In situ synthesis of silver nanoparticles uniformly distributed on polydopamine-coated silk fibers for antibacterial application. J. Colloid Interface Sci..

[15] Annadhasan M., Muthukumarasamyvel T., Sankar Babu V.R., Rajendiran N. (2014). Green Synthesized Silver and Gold Nanoparticles for Colorimetric Detection of Hg^2+^, Pb^2+^, and Mn^2+^ in Aqueous Medium. ACS Sustain. Chem. Eng..

[16] Chandrasekharan D.K., Khanna P.K., Kagiya T.V., Nair C.K.K. (2011). Synthesis of nanosilver using a vitamin C derivative and studies on radiation protection. Cancer Biother. Radiopharm..

[17] Das S., Sharma M., Saharia D., Sarma K.K., Sarma M.G., Borthakur B.B., Bora U. (2015). In vivo studies of silk based gold nano-composite conduits for functional peripheral nerve regeneration. Biomaterials.

[18] Guo C., Hall G.N., Addison J.B., Yarger J.L. (2014). Gold nanoparticle-doped silk film as biocompatible SERS substrate. RSC Adv..

[19] Belda Marín C. (2020). Silk Bionanocomposites: Design, Characterization and Potential Applications. Ph.D. Thesis.

[20] Sangnier A.P., Aufaure R., Cheong S., Motte L., Palpant B., Tilley R.D., Guenin E., Wilhelm C., Lalatonne Y. (2019). Raspberry-like small multicore gold nanostructures for efficient photothermal conversion in the first and second near-infrared windows. Chem. Commun..

[21] Liang B., Yu K., Ling Y., Kolios M., Exner A., Wang Z., Hu B., Zuo G., Chen Y., Zheng Y. (2019). An artificially engineered “tumor bio-magnet” for collecting blood-circulating nanoparticles and magnetic hyperthermia. Biomater. Sci..

[22] Cabana S., Curcio A., Michel A., Wilhelm C., Abou-Hassan A. (2020). Iron Oxide Mediated Photothermal Therapy in the Second Biological Window: A Comparative Study between Magnetite/Maghemite Nanospheres and Nanoflowers. Nanomaterials.

[23] Belda Marín C., Fitzpatrick V., Kaplan D.L., Landoulsi J., Guénin E., Egles C. (2020). Silk Polymers and Nanoparticles: A Powerful Combination for the Design of Versatile Biomaterials. Front. Chem..

[24] Kuhn H., Medlin D. (2000). Mechanical Testing and Evaluation.

[25] Ouellet S., Cronin D., Worswick M. (2006). Compressive response of polymeric foams under quasi-static, medium and high strain rate conditions. Polym. Test..

[26] Rahimidehgolan F., Altenhof W. (2023). Compressive behavior and deformation mechanisms of rigid polymeric foams: A review. Compos. Part B Eng..

[27] Rajput S., Burde H., Singh U.S., Kajaria H., Bhagchandani R.K. (2021). Optimization of prosthetic leg using generative design and compliant mechanism. Mater. Today Proc..

[28] Li B., Aspden R.M. (1997). Composition and mechanical properties of cancellous bone from the femoral head of patients with osteoporosis or osteoarthritis. J. Bone Miner. Res. Off. J. Am. Soc. Bone Miner. Res..

[29] (2009). Biological Evaluation of Medical Devices—Part 5: Tests for In Vitro Cytotoxicity.

[30] Akturk O., Kismet K., Yasti A.C., Kuru S., Duymus M.E., Kaya F., Caydere M., Hucumenoglu S., Keskin D. (2016). Wet electrospun silk fibroin/gold nanoparticle 3D matrices for wound healing applications. RSC Adv..

[31] Tcharkhtchi A., Abdallah-Elhirtsi S., Ebrahimi K., Fitoussi J., Shirinbayan M., Farzaneh S. (2014). Some New Concepts of Shape Memory Effect of Polymers. Polymers.

[32] Du R., Zhao B., Luo K., Wang M.-X., Yuan Q., Yu L.-X., Yang K.-K., Wang Y.-Z. (2023). Shape Memory Polyester Scaffold Promotes Bone Defect Repair through Enhanced Osteogenic Ability and Mechanical Stability. ACS Appl. Mater. Interfaces.

[33] Miao S., Cui H., Esworthy T., Mahadik B., Lee S., Zhou X., Hann S.Y., Fisher J.P., Zhang L.G. (2020). 4D Self-Morphing Culture Substrate for Modulating Cell Differentiation. Adv. Sci..

[34] Mata R., Yao Y., Cao W., Ding J., Zhou T., Zhai Z., Gao C. (2021). The Dynamic Inflammatory Tissue Microenvironment: Signality and Disease Therapy by Biomaterials. Research.

[35] Tang L., Eaton J.W. (1995). Inflammatory responses to biomaterials. Am. J. Clin. Pathol..

[36] Li C., Guo C., Fitzpatrick V., Ibrahim A., Zwierstra M.J., Hanna P., Lechtig A., Nazarian A., Lin S.J., Kaplan D.L. (2020). Design of biodegradable, implantable devices towards clinical translation. Nat. Rev. Mater..

[37] Cohen-Karni T., Jeong K.J., Tsui J.H., Reznor G., Mustata M., Wanunu M., Graham A., Marks C., Bell D.C., Langer R. (2012). Nanocomposite Gold-Silk Nanofibers. Nano Lett..

[38] Sridhar S., Venugopal J.R., Sridhar R., Ramakrishna S. (2015). Cardiogenic differentiation of mesenchymal stem cells with gold nanoparticle loaded functionalized nanofibers. Colloids Surf. B Biointerfaces.

[39] Schneider A., Wang X.Y., Kaplan D.L., Garlick J.A., Egles C. (2009). Biofunctionalized electrospun silk mats as a topical bioactive dressing for accelerated wound healing. Acta Biomater..

[40] Pritchard E.M., Hu X., Finley V., Kuo C.K., Kaplan D.L. (2013). Effect of Silk Protein Processing on Drug Delivery from Silk Films. Macromol. Biosci..

[41] Balfourier A., Luciani N., Wang G., Lelong G., Ersen O., Khelfa A., Alloyeau D., Gazeau F., Carn F. (2020). Unexpected intracellular biodegradation and recrystallization of gold nanoparticles. Proc. Natl. Acad. Sci. USA.

[42] Benyettou F., Prakasam T., Nair A.R., Witzel I.-I., Alhashimi M., Skorjanc T., Olsen J.-C., Sadler K.C., Trabolsi A. (2019). Potent and selective in vitro and in vivo antiproliferative effects of metal–organic trefoil knots. Chem. Sci..

[43] Qian K.-Y., Song Y., Yan X., Dong L., Xue J., Xu Y., Wang B., Cao B., Hou Q., Peng W. (2020). Injectable ferrimagnetic silk fibroin hydrogel for magnetic hyperthermia ablation of deep tumor. Biomaterials.

[44] Santos L.J., Reis R.L., Gomes M.E. (2015). Harnessing magnetic-mechano actuation in regenerative medicine and tissue engineering. Trends Biotechnol..

[45] Aufaure R., Buendia R., Motte L., Hardouin J., Lalatonne Y., Guénin E. (2017). Versatile “click” synthesis of 1-hydroxy-1,1-methylenebisphosphonic acids with thioalkoxy substituents for the preparation of stable gold nanoparticles. New J. Chem..

[46] Belkahla H., Antunes J.C., Lalatonne Y., Catherine O.S., Illoul C., Journé C., Jandrot-Perrus M., Coradin T., Gigoux V., Guenin E. (2020). USPIO–PEG nanoparticles functionalized with a highly specific collagen-binding peptide: A step towards MRI diagnosis of fibrosis. J. Mater. Chem. B.

[47] Pang Z., Chong J., Zhou G., de Lima Morais D.A., Chang L., Barrette M., Gauthier C., Jacques P.-É., Li S., Xia J. (2021). MetaboAnalyst 5.0: Narrowing the gap between raw spectra and functional insights. Nucleic Acids Res..

[48] The European Parliament, Council of the European Union (2010). Directive 2010/63/EU of the European Parliament and of the Council.

[49] Percie du Sert N., Hurst V., Ahluwalia A., Alam S., Avey M.T., Baker M., Browne W.J., Clark A., Cuthill I.C., Dirnagl U. (2020). The ARRIVE guidelines 2.0: Updated guidelines for reporting animal research. PLoS Biol..

[50] (2016). Biological Evaluation of Medical Devices—Part 6: Tests for Local Effects After Implantation.

